# Effect of femoral head size on the wear of metal on metal bearings in total hip replacements under adverse edge-loading conditions

**DOI:** 10.1002/jbm.b.32824

**Published:** 2012-12-20

**Authors:** Mazen Al-Hajjar, John Fisher, Sophie Williams, Joanne L Tipper, Louise M Jennings

**Affiliations:** 1 Institute of Medical and Biological Engineering, School of Mechanical Engineering, University of LeedsLeeds LS2 9JT, United Kingdom; 2 Leeds Musculoskeletal Biomedical Research Unit, Leeds Teaching Hospital Trust, University of LeedsLeeds LS2 9JT, United Kingdom

**Keywords:** edge loading, metal-on-metal, hip replacement, microseparation, inclination angle

## Abstract

Metal-on-metal (MoM) bearings have shown low-wear rates under standard hip simulator conditions; however, retrieval studies have shown large variations in wear rates and mechanisms. High-wear *in vivo* has caused catastrophic complications and has been associated with steep cup-inclination angle (rotational malpositioning). However, increasing the cup-inclination angle *in vitro* has not replicated the increases in wear to the same extent as those observed in retrievals. Clinically relevant wear rates, patterns, and particles were observed *in vitro* for ceramic-on-ceramic bearings when microseparation (translational malpositioning) conditions were introduced into the gait cycle. In the present study, 28 and 36-mm MoM bearings were investigated under adverse conditions. Increasing the cup angle from 45° to 65° resulted in a significant increase in the wear rate of the 28 mm bearings. However, for the 36 mm bearings, head-rim contact did not occur under the steep cup-angle condition, and the wear rate did not increase. The introduction of microseparation to the gait cycle significantly increased the wear rate of the MoM bearings. Cup angle and head size did not influence the wear rate under microseparation conditions. This study indicated that high-*in vivo* wear rates were associated with edge loading due to rotational malpositioning such as high-cup-inclination angle and translational malpositioning that could occur due to several surgical factors. Translational malpositioning had a more dominant effect on the wear rate. Preclinical simulation testing should be undertaken with translational and rotational malpositioning conditions as well as standard walking cycle conditions defined by the ISO standard. © 2012 Wiley Periodicals, Inc. J Biomed Mater Res Part B: Appl Biomater, 2013.

## INTRODUCTION

Metal-on-metal (MoM) bearings in total hip replacements (THRs) have been used as an alternative to metal-on-polyethylene due to the polyethylene particles inducing osteolysis.^1^ One retrieval analysis of MoM bearings has reported a low-steady-state wear rate of 5 μm/year.^2^ However, more recent retrieval studies of MoM bearings in THRs have shown a wide range of clinical wear rates^3^ and wear mechanisms.^4^ High-wear rates measured on retrievals, including surface replacements (SRs), have been widely reported.^3^, ^5^, ^6^

MoM bearings in THRs have shown low-*in vitro* wear rates under standard hip simulator conditions, which correlate with well-positioned prostheses.^7^–^9^ Under these standard conditions, the centers of rotation of the femoral head and the acetabular cup are matched, and the inclination angle of the acetabular cup is below a clinical equivalent of 55°. With these conditions, the contact area (wear patch) is within the intended bearing surface, and mixed lubrication regimes are dominant. The wear is split into two phases, an initial bedding in phase and then a steady-state phase with a lower wear rate.

High-wearing MoM SR retrievals have been associated with steep cup-inclination angle.^6^, ^10^ Also, high levels of metal ions have been measured in patients with steeply inclined metal acetabular components.^11^*In vitro* studies have shown that increasing the cup-inclination angle of MoM bearings resulted in edge loading and elevation of wear rates^12^–^14^; although these do not generally reach the high levels observed in some retrievals.

Second-generation alumina ceramic-on-ceramic (CoC) retrieval studies have shown a stripe-like wear area on the femoral head and have associated increased wear rates with high-cup-inclination angle.^15^, ^16^*In vitro* studies have shown that increasing the cup-inclination angle on its own did not replicate *in vivo* wear rates and wear mechanisms^17^; however, the introduction of microseparation translational malpositioning to the gait cycle resulted in edge loading and wear rates, wear mechanisms, and bimodal nano- and micron-sized wear particles similar to those seen in retrievals.^18^–^20^ Microseparation was provided during the swing phase of the gait cycle by positioning the femoral head laterally relative to the acetabular cup. Edge loading occurred at heel strike, producing a stripe of wear on the femoral head with a corresponding wear area on the acetabular cup, as the head moved to a concentric position with the cup through the remainder of the stance phase.

In a well-positioned prosthesis, the centers of rotation of the femoral head and the acetabular cup are matched. Microseparation conditions occur when these centers of rotation are separated, which may also be described as translational malpositioning. If the level of separation exceeds the radial clearance of the bearing couple, edge loading may occur. Surgical procedures aim to restore the joint function by accurate component positioning and the correct soft-tissue tension, which includes correct leg length and offset and avoiding impingement of the femoral neck with the acetabular cup.^21^–^24^ Incorrect joint center or soft-tissue tension may lead to mismatch in the centers of rotation of the acetabular cup and the femoral head. Thus, microseparation conditions may occur due to several clinical factors such as head-offset deficiency, medialized cup, stem subsidence, impingement, and laxity of the soft tissues resulting in a translational misalignment of the femoral head and acetabular cup.^25^ It should be noted that while rotational misalignment such as variation in cup position is readily detected radiographically, translational misalignment is not. If components are translationally malpositioned surgically with respect to the anatomical centers of the femur and pelvis, when the hip joint is assembled, then radiographically, the head is located within the confines of the cup. The head is in effect constrained by the rim of the cup, so that the center of the head is separated from the center of the cup by the small radial clearance between the head and cup, which cannot be detected on a radiograph. Nevelos et al.^15^ have shown a strong correlation between stripe wear and increased cup-inclination angle, suggesting that a steeply inclined acetabular cup could also facilitate the occurrence of translational malpositioning and microseparation conditions. These microseparation simulator conditions, which have been uniquely validated against CoC retrievals, were introduced into the gait cycle in MoM studies, and several studies showed a significant increase in wear rate, formation of stripe wear on the femoral head with rim wear on the acetabular cups and micron sized as well as nanometer-sized wear particles.^14^, ^26^

High-wear rates of MoM bearings have been associated with many clinical complications that lead to complicated revision surgeries. Aseptic lymphocyte dominated vasculitis associated lesions, pseudotumors, pain, and osteolysis are consequences of high-wearing MoM bearings.^10^, ^27^–^29^ Fluoroscopy studies have shown that microseparation occurs in hip-joint replacement.^30^, ^31^ Also, a recent retrieval study of 120 MoM SRs and 120 MoM modular THRs has indicated that edge-loading conditions were common in 67% of retrieved hip resurfacing bearings and in 57% of retrieved modular THR bearings.^32^ Kwon et al.^10^ have shown a positive correlation between the development of pseudotumors in patients with MoM bearings and the occurrence of edge-loading conditions. This confirms the necessity of introducing edge-loading and microseparation conditions^18^ to standard gait cycles when testing MoM bearings in *in vitro* hip-simulator studies.

Williams et al.^12^ examined the effect of microseparation on the wear of size 28 MoM THRs. The study showed a sixfold increase in the wear rate under microseparation conditions compared to the steady-state wear rates obtained under standard gait conditions. Williams et al.^12^ generated microseparation by applying a small negative force during the swing phase causing an inferior lateral movement of the femoral head relative to the cup. The femoral head contacted the inferior rim of the acetabular cup before edge loading occurred at heel strike. Leslie et al.^14^ tested size 39-mm MoM SR bearings under microseparation conditions using the method described by Nevelos et al.^18^ and reported a 14-fold increase in wear rates compared to steady-state wear rates under standard gait-cycle conditions. Leslie et al.^14^ obtained wear rates similar to those observed in retrievals.^6^ Although the wear rate of size 39-mm SR bearings showed a higher increase than that of size 28-mm THRs, there were many factors that may have contributed to the difference such as diametrical clearance, head size, prosthesis design especially acetabular rim radius, and testing method.

It has been shown that steeply inclined acetabular cups or microseparation conditions can cause higher wear rates and consequently reduce prosthesis lifetime; however, the relative contribution to the increased wear of these implants under different adverse conditions and the influence of head size are not fully understood. The aim of this study was to systematically investigate the influence of head size of MoM bearings in THRs under adverse clinically relevant hip simulator conditions. The relative contributions to wear of increased cup-inclination angle and microseparation conditions were investigated by testing both conditions independently and in combination.

## MATERIALS AND METHODS

The wear of MoM bearings in THRs was investigated under different cup-inclination angles during standard gait and microseparation conditions. Six 28 mm and six 36 mm diameter cobalt chrome alloy femoral heads and acetabular cups were custom-manufactured by Corin (Cirencester, UK). The components were all high-carbon alloys [>0.2% (w/w) carbon], and all components were heat-treated. All bearings couples had a diametrical clearance that ranged between 40 and 60 μm (Table I). The acetabular cups used with both head sizes had an outer diameter of 54 mm, a coverage angle of 160°, and a rim radius of 0.5 mm.

**TABLE I:** The Sizes and Clearances of the Components used in this Study

	28-mm bearings couples			36-mm bearings couples		
		
	Head Diameter (mm)	Cup Inner Diameter (mm)	Diametrical Clearance (mm)	Head Diameter (mm)	Cup Inner Diameter (mm)	Diametrical Clearance (mm)
Station 1	27.947	27.988	0.041	35.906	35.952	0.046
Station 2	27.971	28.011	0.040	35.920	35.960	0.040
Station 3	28.028	28.068	0.040	35.906	35.963	0.057
Station 4	27.981	28.021	0.040	35.925	35.965	0.040
Station 5	27.985	28.028	0.043	35.933	35.973	0.040
Station 6	27.985	28.025	0.040	35.907	35.957	0.051

The bearings were studied in the six station Leeds II Physiological Anatomical Hip Joint simulator. A twin peak loading of 3 kN peak load was applied, and two independently controlled axes of motion, extension/ flexion (−15° to +30°), and internal/external rotation (±10°) were applied.

For each bearing size, three acetabular cups were mounted at an angle equivalent to 45° clinical cup-inclination angle, and the remaining three were mounted at an angle equivalent to 65° clinically. The cups were mounted directly in the cup holders using PMMA resin providing uniform support from the back to avoid any deformation. A keyway was machined on the back of the cup to avoid rotation in the cup holder. Stainless-steel locking rings were used to hold the cups in place. The setup was performed, so that the cups could be easily removed from the cup holders for measurement. The first 3 million cycles were performed under standard gait conditions. The “severe” microseparation conditions, described in previous studies,^18^, ^33^ were introduced to all six stations for the subsequent 3 million cycles. The microseparation condition was achieved by applying a lateral movement of ∼0.5 mm to the acetabular cup relative to the head, which resulted in edge loading at the rim of the cup at heel strike. This loading regime has previously been shown to replicate clinically relevant wear rates, mechanisms, and debris in CoC prostheses^18^ and has been used in several studies.^14^, ^18^, ^26^, ^33^–^37^ The setup allowed comparison between four different testing conditions summarized in Table II. The lubricant used was 25% (v/v) new-born calf serum supplemented with 0.03% (v/v) of sodium azide to inhibit bacterial growth. The protein concentration in the 25% (v/v) serum used was ∼15 g/L. A volume of 450 mL was used in each serum bath of each station and changed every 333,000 cycles. Wear measurements were undertaken every 1 million cycles. The wear volume was ascertained through gravimetric analysis. The components were weighed using a Mettler AT201 balance (Leicester, UK; 0.01 mg resolution).^8^

**TABLE II:** The Four Simulator Test Conditions Investigated in this Study

	Cup-Inclination Angle	
	
	45°	65°
Standard gait cycle	Standard conditions	Steep cup-inclination angle conditions
Microseparation introduced to gait cycle	Microseparation conditions	Steep cup-inclination angle under microseparation conditions

Cobalt and chromium serum ion concentrations were measured throughout the test using inductively coupled plasma–mass spectrometry (Lambda Physik, Göttingen, Germany). The serum was centrifuged to eliminate the wear debris, and nitric acid was used to digest the proteins present in the serum. Throughout each test, for each condition investigated, 450 mL of serum in each station was collected every 330,000 cycles over the entire 3 million cycles of the test. The serum samples between 1 and 2 million cycles and 2 and 3 million cycles were pooled. The ion concentration measurement points were then as follows: 0–330,000 cycles, 333,000–666,000 cycles, 666,000–1 million cycles, 1–2 million cycles, and 2–3 million cycles.

A two-dimensional contacting profilometer (Form Talysurf series, Taylor Hobson, UK) was used to measure the surface roughness of the components before and after testing. The penetration depth over the wear stripe produced due to edge loading was measured. Three traces were taken across the wear scar 5 mm apart. A scanning electron microscope (SEM, Philips XL30) was used to take high-magnification images over the wear area of the femoral head and acetabular cups. Statistical analysis was performed using one-way ANOVA (significance taken at *p* < 0.05), and 95% confidence limits were calculated.

## RESULTS

**FIGURE 1:**
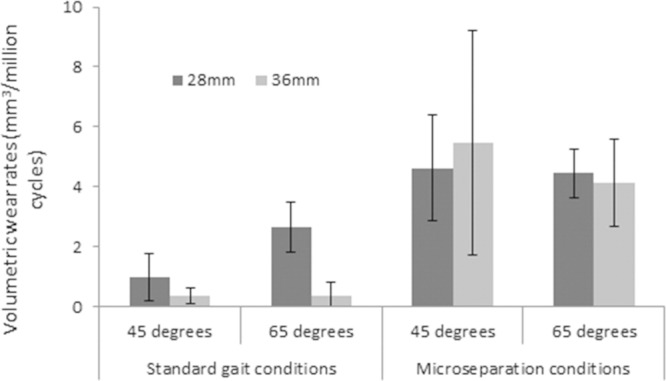
Mean wear rates under the four test conditions (*n* = 3). Error bars represent 95% confidence limit.

**FIGURE 2:**
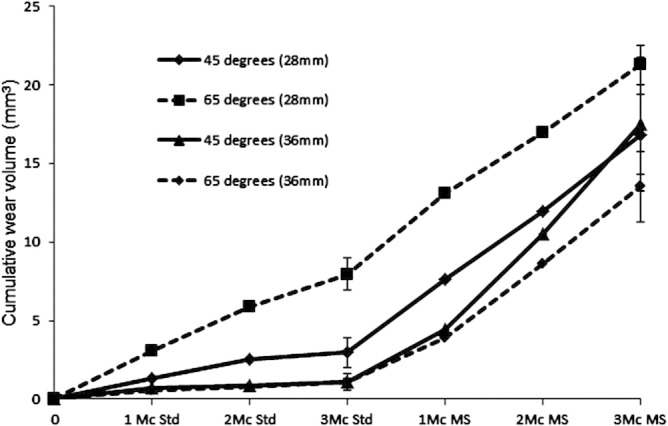
Mean cumulative wear volume over the 6 million cycles (Mc) of testing (*n* = 3) under standard (Std) and microseparation (MS) conditions. Error bars represent ± one standard deviation.

Under standard gait conditions, the wear rate of the smaller 28-mm MoM bearings significantly (*p* < 0.01) increased from 0.99 to 2.65 mm^3^/million cycles when the cup-inclination angle was increased from 45° to 65° (Figure 1). However, for the 36-mm bearings, there was no statistically significant difference in the wear rates obtained under standard (45°) and steep (65°) cup-inclination angle conditions (0.35 and 0.37 mm^3^/million cycles for 45° and 65° cup-inclination angles, respectively; *p* = 0.9). For the 28-mm bearings, the introduction of microseparation conditions to the gait cycle caused a large and significant increase in the wear rate to 4.62 mm^3^/million cycles for a cup-inclination angle of 45° (*p* < 0.01) and to 4.44 mm^3^/million cycles for a cup-inclination angle of 65° (*p* < 0.01; Figure 1). For the 36-mm bearings, the introduction of microseparation conditions to the gait cycle also produced a large increase in the wear rate to 5.47 mm^3^/million cycles for a cup-inclination angle of 45° (*p* < 0.01) and to 4.14 mm^3^/million cycles for a cup-inclination angle of 65° (*p* < 0.01; Figure 1). However, increasing the cup-inclination angle from 45° to 65° under microseparation conditions did not cause a statistically significant (*p* = 0.7) change in the wear rate of either size of MoM bearing (Figure 1). There was no statistically significant change in the wear rate under microseparation conditions throughout the 3 million cycles of testing (when comparing 0–1 million cycles, 1–2 million cycles, and 2–3 million cycles wear rates under microseparation conditions), regardless of the cup-inclination angles and bearing size (Figure 2).

**FIGURE 3:**
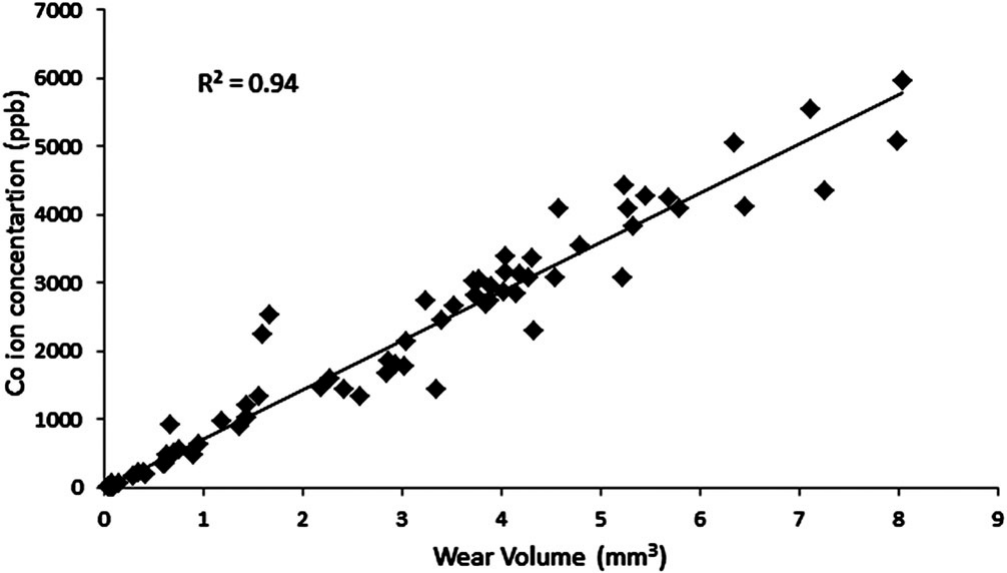
Correlation between cobalt ion concentration in serum and wear volume at each measurement point for both MoM-bearing sizes.

**FIGURE 4:**
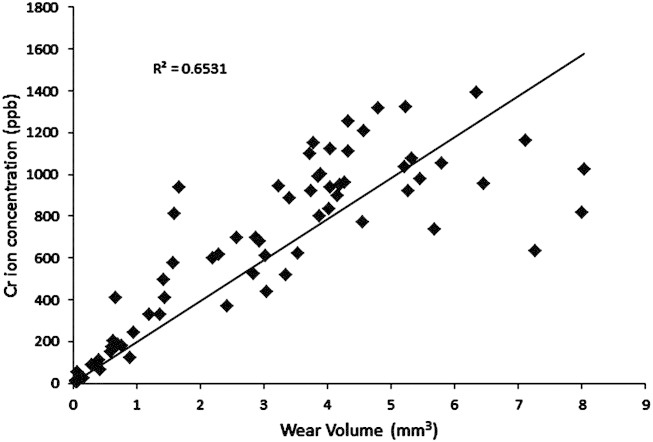
Correlation between chromium ion concentration in serum and wear volume at each measurement point for both MoM-bearing sizes.

Cobalt ion concentration measured in the serum collected from each station throughout the hip simulator tests for both bearing sizes showed a strong correlation (*R*^2^ = 0.94) with the wear volumes measured using gravimetric analysis (Figure 3). However, for chromium ions, the correlation was weaker (*R*^2^ = 0.65) especially for higher wear volumes above 5 mm^3^ (Figure 4).

A stripe of wear on the metal heads with a corresponding wear area on the superior lateral edge of the acetabular cup was observed when microseparation conditions were introduced to the gait cycle. For the 28-mm bearings, the mean maximum penetration depth on the femoral heads was 57 μm under the standard cup-inclination angle condition and 74 μm under the steep cup-inclination angle condition. There was no significant difference (*p* = 0.22) in the penetration depths between the two cup-inclination angle conditions. For the 36-mm bearings, the mean penetration depth on the femoral heads was 55 μm under the standard cup-inclination angle condition and 48 μm under the steep cup-inclination angle condition. There was no significant difference (*p* = 0.44) in the penetration depths between the two cup-inclination angle conditions.

**FIGURE 5:**
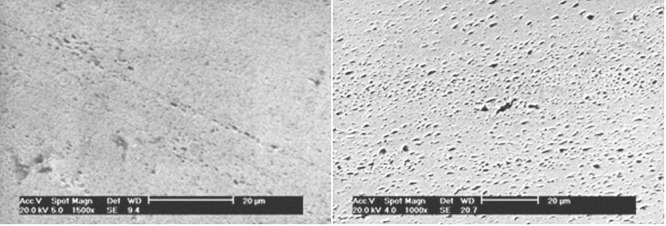
Example of surface texture over the wear area of the femoral head (left, 1500× magnification) and the acetabular cup (right, 1000× magnification) under standard conditions after 3 million cycles of test. Micropitting was a dominant feature over the wear area.

**FIGURE 6:**
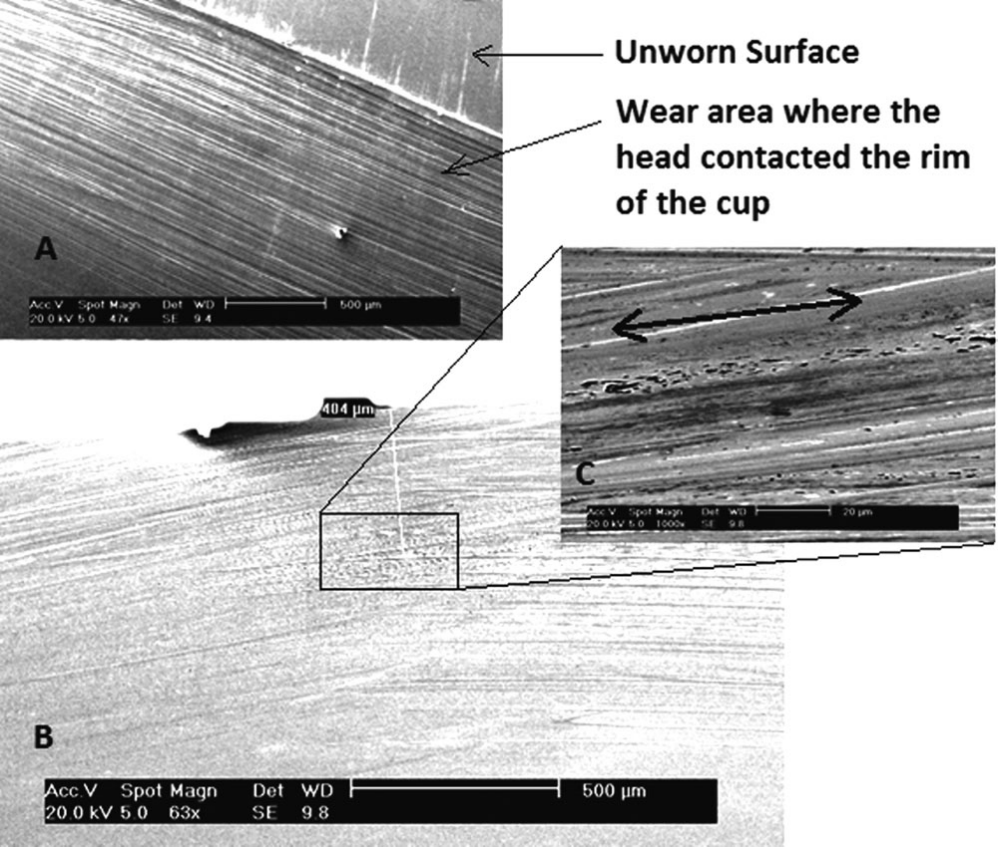
Surface damage on the femoral head (A) and near the rim of the acetabular cup (B) under the steep cup-inclination angle condition. A: Low magnification image (×47) showing the scratches on the femoral head due to head-rim contact. B, C: Low and high-magnification images respectively showing the detailed texture of the surface near the rim of the cup. Double-sided arrow shows the orientation of the acetabular rim.

**FIGURE 7:**
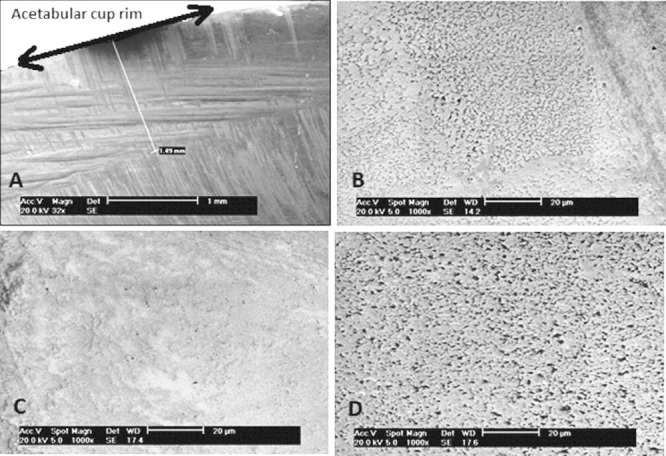
Surface damage near the rim of the cup under microseparation conditions. A: Low magnification (×32) showing the scratches near the rim of the cup; scratches are in different directions. B–D: High-magnification images showing detailed textures of the surface damage. A range of wear patterns were observed on the surface under microseparation conditions. A: Double-sided arrow shows the orientation of the acetabular rim.

**FIGURE 8:**
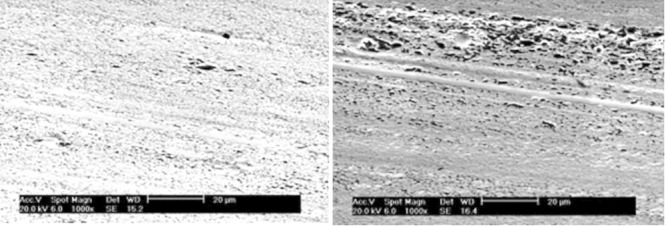
Examples of surface damage near the rim of the cup under microseparation conditions with a steeply inclined cup, showing elongated pits and scratches along the rim of the cup (1000× magnifications).

Under standard conditions, high-magnification SEM images showed evidence of micropitting over the wear area of the head and the cup (Figure 5). Under edge-loading conditions, the wear area, where the head contacted the rim of the cup, became rougher. On the femoral head, the *R*_a_ increased from a range of 29–50 to 50–115 nm, and on the acetabular cup, the *R*_a_ increased from a range of 16–33 to 40–95 nm. Under steep-cup-inclination angle conditions, high-magnification images near the rim of the cup showed scratches along the rim with elongated pits of different sizes (Figure 6). Under microseparation conditions, there were several wear patterns such as abrasive wear, micropitting, and polishing of the surface (Figures 7 and 8).

## DISCUSSION

High-wear rates^6^, ^11^ and ion levels^11^ in patients with MoM bearings have been associated with steep acetabular cup-inclination angles. However, *in vitro* studies with steeply inclined acetabular cups^13^, ^26^ have not replicated the level of increase in wear rates that have been observed in retrievals. This indicates that other conditions or factors are influencing or causing the high levels of wear *in vivo*. *In vitro* studies with CoC bearings where microseparation conditions were introduced to the gait cycle leading to edge loading have replicated *in vivo* wear rates, wear mechanisms, and bimodal nano- and micron-sized wear particles.^16^, ^18^–^20^ Fisher^25^ explained the adverse conditions that may produce edge loading and increased wear. These could occur due to either rotational or translational malpositioning of the bearing couple. Rotational malpositioning of the acetabular cup resulting in excessive inclination or version angles causes the femoral head to contact the acetabular cup rim. Translational malpositioning where the centers of the head and the cup are displaced relative to one another (microseparation) can occur due to several reasons such as head offset deficiency, medialized cup, stem subsidence, impingement, and laxity of the soft tissues. Microseparation conditions do not necessarily mean a physical separation of the surfaces of the head and the acetabular cup but a translational displacement between the centers of the head and the cup higher than the radial clearance (<0.5 mm). This study aimed to investigate the influence of head size under adverse rotational malpositioning (increased cup-inclination angle) and translational malpositioning (microseparation conditions) independently and in combination and to assess their relative contributions to increasing the wear of the implants under these different conditions.

Under standard gait conditions when the cup-inclination angle was at 45°, the wear rate was split into two phases, a bedding in phase and a steady-state phase.^38^ In this study, the steady-state wear rate for the 28 mm bearings was 0.44 mm^3^/million cycles, which is consistent with previous studies for 28 mm bearings, tested with a 300N ISO standard swing phase load.^26^, ^39^ Ion-level analysis showed a similar pattern to the wear results, with the steady-state phase reached between 1 and 2 million cycles. As the serum collected between 1 and 2 million cycles was pooled together, it was not possible to determine at what point between 1 and 2 million cycles the steady-state phase was reached. The larger size bearings (36 mm) generated a significantly lower steady-state wear rate than the 28 mm bearings (0.17 mm^3^/million cycles), which was again consistent with previous studies.^40^–^43^ The wear results also showed bedding in and steady-state phases where the steady-state phase occurred at an earlier point for the 36 mm bearing compared to the 28 mm bearings, between 0 and 1 million cycles. It was not possible to determine when exactly the steady-state was reached between 0 and 1 million cycles from the wear data, as the first wear measurement point was not taken until 1 million cycles of testing were completed. However, the first ion-level measurement point was at 330,000 cycles followed by a measurement at 660,000 cycles. There was a sharp drop in ion concentration between 330,000 and 660,000 cycles, indicating that the steady-state phase was reached at a point between 0 and 330,000 cycles.

In this study, the wear rates obtained under standard conditions were lower than previously reported for the 36-mm MoM bearings.^44^ This could be due to the reduced diametrical clearance used in this study (40 μm) compared to ∼80 μm in the previous study. Farrar and Schmidt^45^ have shown that decreasing the diametrical clearance reduces the bedding in wear of MoM articulations.

**FIGURE 9:**
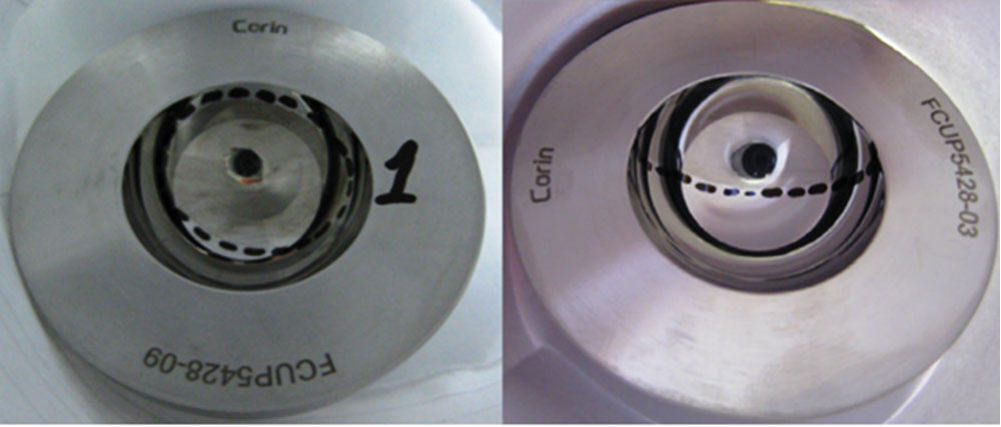
Photographs of the 28-mm inner diameter acetabular cups showing the wear area (within dotted line) under standard conditions with 45° inclination angle (left) and the wear area under steep inclination angle (right). Under steep inclination angle, the wear area intersected with the rim of the acetabular cup. [Color figure can be viewed in the online issue, which is available at wileyonlinelibrary.com.]

When the cup-inclination angle was increased to 65°, the contact area between the head and the cup, for the 28-mm bearings, decreased and migrated toward the rim resulting in the head contacting the superior rim of the acetabular cup (Figure 9) and resulting in high-contact stresses, reduction in lubrication, and hence an increased wear rate and no evidence of a steady-state phase after 3 million cycles. The wear rates obtained in this study under steep cup-inclination angle were of the same order of magnitude to the bedding in wear rates of some MoM SRs previously tested and reported under standard conditions.^26^, ^42^, ^44^ Under standard conditions, the bedding in period only lasted up to the first 1 or 2 million cycles, and then a low-steady state wear rate was reached. However, under edge-loading conditions, due to steep-inclination angles, there was no sign of a low-steady state phase and the wear rate remained linear throughout the entire test. For the standard condition testing, when the 45° cups reached steady-state wear between 2 and 3 million cycles, there was a 4.6-fold increase in the wear rate between 2 and 3 million cycles when the cup-inclination angle was increased to 65°. This was consistent with previous studies that showed increased wear rates in MoM bearings with increased cup-inclination angle.^13^, ^14^, ^26^ The relative increases in wear could have been influenced by the different prosthesis designs used, especially the acetabular cup rim profile, as well as cup-inclination angle. Another study on 39-mm MoM SR showed a ninefold increase in the wear rate when the cup angle was increased from 45° to 60°.^14^ The SR cup used was larger in size and had a smaller inclusion angle and a different rim profile than the metal cup used in the current study, which again may have contributed to the higher wear rates obtained here under steeply inclined cup conditions.

**FIGURE 10:**
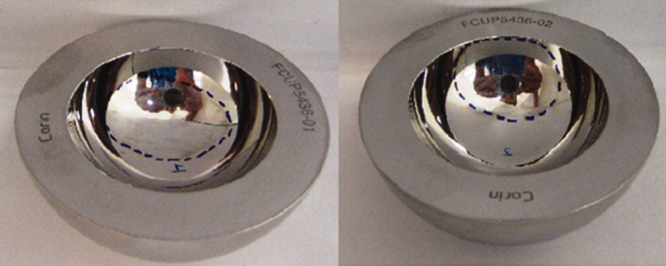
Photographs of the 36-mm inner diameter acetabular cups showing the wear area (within dotted line) under standard conditions with 45° inclination angle (left) and the wear area under steep inclination angle (right). Under steep inclination angle, the wear area approached the rim of the acetabular cup, but it did not intersect with the rim after 3 million cycles of test under standard gait conditions. [Color figure can be viewed in the online issue, which is available at wileyonlinelibrary.com.]

The 36-mm bearings showed no increase in wear rate when the cup-inclination angle was increased from 45° to 65°. As the inclination angle increased, the contact area approached the rim of the acetabular cup but no head-rim contact occurred (Figure 10). Both bearing sizes used in this study had an inclusion angle (cup coverage) of 160°. A cup designed with a hemispherical inclusion angle (180°) will have better tolerance to rotational malpositioning; however, this will restrict the range of motion and increase the incidence of impingement.^46^ Decreasing the acetabular coverage will increase the range of motion but will increase the chance of edge loading due to rotational malpositioning. These results shows that increased wear due to rotational malpositioning only occurs when edge loading occurs, which in turn is dependent on the combination of several factors such as steep cup-inclination angles, excessive version or anteversion angles, and acetabular cup geometries and component size.

When microseparation conditions were introduced to the gait cycle, there were significant increases in wear rates for both bearings sizes, and a stripe of wear was formed on the femoral head with a corresponding wear area at the superior rim of the acetabular cup. The wear rate throughout the 3 million cycles of testing under microseparation conditions for both bearing sizes was steady, showing no evidence of bedding in and steady-state phases. The results showed no statistically significant differences in the wear rate between the two cup-inclination angle conditions under microseparation conditions, indicating that edge loading due to microseparation conditions dominates the effect of head-rim contact due to steeply inclined acetabular cups.

Under standard conditions, the wear rates decreased with increasing head size due to improved lubrication regimes. However, when edge loading occurred under microseparation conditions, the wear rate of the 36-mm bearings was similar to the 28-mm bearings. It is postulated that this was due to a change in the lubrication regime from mixed lubrication, which gave the larger bearings their superior wear resistance under standard gait conditions to boundary lubrication. The wear rate obtained for both bearing sizes under microseparation and edge-loading conditions in this study was lower than that reported previously for 39-mm MoM SR bearings tested under similar conditions.^14^ This difference may be due to the different prosthesis design, in particular the inclusion angle of the cup or the rim design. The previous *in vitro* study, which tested 39-mm MoM SR under microseparation conditions,^14^ showed comparable wear values to retrieved SR bearings that had experienced edge-loading conditions.^6^ It is clear from the literature that there is a wide range of wear rates observed *in vivo*, as well as cup position, inclination, and version, soft tissue tension, impingement, and microseparation may affect wear rates. But, in addition, under adverse rim-loading conditions, other design factors such as rim geometry and cup-inclusion angle may also impact on the increase in wear for different designs.

Serum cobalt ion concentrations measured in this study showed a strong correlation with the wear volumes measured gravimetrically. However, chromium ion concentrations showed a weaker correlation with wear volume, especially at high-wear volumes. Under microseparation and edge-loading conditions, MoM bearings produce micrometer-sized particles as well as nanometer-sized particles.^14^ These relatively large particles are rich in chromium oxide and are removed by centrifuging when preparing samples for ion-level measurement.^47^ This could explain the lower-than-expected chromium ion levels at high-wear volumes, which were obtained under edge-loading conditions.

Surface analysis indicated two distinct wear areas under adverse simulator conditions: a wear area similar to that obtained under standard gait-cycle conditions and another wear area where the head contacted the rim of the cup under edge-loading conditions. Under steep cup-inclination angle conditions, the contact between the head and the rim of the cup resulted in a stripe of wear featuring elongated micropits along the rim with scratches running alongside. Under microseparation and edge-loading conditions, however, the stripe of wear showed a large variation in the wear features including abrasive wear, micropitting, polishing, and multidirectional scratches.

CoC bearings, unlike MoM bearings, showed no increase in wear due to head-rim contact under increased cup-inclination angle.^17^, ^34^ However, microseparation and edge-loading conditions resulted in stripe wear and increased wear in CoC bearings.^17^, ^33^–^37^, ^48^ For 28-mm BIOLOX® Delta CoC bearings, the wear rate under microseparation conditions was 0.12-mm^3^/million cycles; ∼40 times lower than the wear rate of metal on metal bearings under the same conditions using the same simulator.^34^ The mean penetration over the wear stripe on the ceramic femoral heads over 3 million cycles of testing was below 8 μm^34^ compared to a mean of ∼65 μm for the same-sized MoM bearings tested in this study.

The results of this study suggest that high-wear rate and variations in the wear mechanisms are influenced by edge loading due to microseparation. *In vivo*, steep cup-inclination angles and other factors such as impingement, stem subsidence, tissue laxity around the prosthesis, and head position could facilitate microseparation and edge loading leading to various complications and implant failure. This study highlights the importance of prosthesis design and the accurate positioning of the implant in its optimum position during surgery. The full range of conditions, which generate malpositioning *in vivo*, rotational and translational malpositioning have not been investigated, and future work will study effects of other variations in positioning such as version, the effects of different activities, which can cause edge loading, as well as investigations of other design variables.
